# Intelligent Drone Swarms to Search for Victims in Post-Disaster Areas

**DOI:** 10.3390/s23239540

**Published:** 2023-11-30

**Authors:** Matheus Nohra Haddad, Andréa Cynthia Santos, Christophe Duhamel, Amadeu Almeida Coco

**Affiliations:** 1 LITIS, ISEL, Université Le Havre Normandie, 25 Rue Philippe Lebon, 76600 Le Havre, France; andrea-cynthia.duhamel@univ-lehavre.fr (A.C.S.); christophe.duhamel@univ-lehavre.fr (C.D.); amadeu.almeidacoco@univ-lille.fr (A.A.C.); 2 CRP-IEP, Universidade Federal de Viçosa, Km 7, MG-230, Rodoviário, Rio Paranaíba 38810-000, Brazil

**Keywords:** drone swarms, routing, multi-agents systems, humanitarian logistics, disaster relief

## Abstract

This study presents the Drone Swarms Routing Problem (DSRP), which consists of identifying the maximum number of victims in post-disaster areas. The post-disaster area is modeled in a complete graph, where each search location is represented by a vertex, and the edges are the shortest paths between destinations, with an associated weight, corresponding to the battery consumption to fly to a location. In addition, in the DSRP addressed here, a set of drones are deployed in a cooperative drone swarms approach to boost the search. In this context, a V-shaped formation is applied with leader replacements, which allows energy saving. We propose a computation model for the DSRP that considers each drone as an agent that selects the next search location to visit through a simple and efficient method, the Drone Swarm Heuristic. In order to evaluate the proposed model, scenarios based on the Beirut port explosion in 2020 are used. Numerical experiments are presented in the offline and online versions of the proposed method. The results from such scenarios showed the efficiency of the proposed approach, attesting not only the coverage capacity of the computational model but also the advantage of adopting the V-shaped formation flight with leader replacements.

## 1. Introduction

After a major disaster, it is very likely that access to some areas can be extremely difficult due to the damage caused by the disaster itself, road congestion or blockages, or even the contamination of dangerous products. This was the case of the Beirut port explosion in Lebanon in 2020 that caused several causalities, more than 7000 injured people, and left about 30,000 homeless [1]. In this context, an alternative strategy to circumvent the aforementioned accessibility difficulties is to use drones to perform tasks such as the distribution of drugs and food, the detection of ground conditions, and to search for victims. They can also quickly access hard-to-reach areas. An extensive study in [2] demonstrates the capabilities, performance outcomes and barriers of using drones in the context of humanitarian logistics.

In such a context, the search for victims is a crucial operation and must be well-planned and carried out with extreme efficiency to save lives. The search for victims can be supported by the use of drones. Moreover, the search can be boosted by using a fleet of drones equipped with optical and thermal cameras, flying in a coordinate swarm. This allows them to share updated information about the area, to be more efficient in scanning the area, and also to save energy using a special flight organization. As shown in [3], energy consumption can be reduced by applying a V-shaped formation with leader replacements, which is inspired by bird flight.

The search for victims using drone swarms involves not only solving a path planning problem [4], but also solving an optimization problem for finding the route that minimizes/maximizes a given criterion, which is the main focus of this study. More specifically, this study proposes the Drone Swarms Routing Problem (DSRP) and consists of defining routes for drone swarms in order to maximize the number of victims identified. The novelties of the DSRP are the consideration of the cooperative and decentralized flight of drone swarms, where they decide on the fly if they scan a location together or not. The interest of using such a strategy is to cover faster an area and to save energy, by means of an appropriate swarm organization. Civil drones still have low autonomy, which makes scanning large areas difficult. Moreover, the hydrogen technology for drones is not yet operational. This study extends the work of [5], in which, drones fly independently of each other with routes predefined by a control center.

A computational model to solve the DSRP is proposed here, using the concept of Multi-Agents Systems (MAS), where drones are agents able to analyze information about the post-disaster area, and smartly select the next search location to perform the search for victims. For such a purpose, a Drone Swarm Heuristic (DSH) is applied to carry out the decision step of the drones. The proposed method is tested over a case study inspired by the Beirut port explosion in 2020. Numerical experiments considering offline and online versions of the method are presented. In the former, the input data are predefined, while in the latter, data changes during the execution of the model. The DSRP solutions produced by the proposed method are also observed in the robot simulator CoppeliaSim [6] to simulate their execution step-by-step.

This study makes several contributions, in particular, we propose:

- the new DSRP to search for victims in post-disaster scenarios;
- a computational model based on multi-agent systems;
- an offline and online heuristics for the DSRP;
- solutions are tested using the CoppeliaSim simulator;
- an adaptation of instances proposed in [5] for DSRP.

The remainder of this paper is organized as follows. The Drone Swarms Routing Problem is defined in Section 2. In Section 3, closely related studies are presented. The proposed DSRP computational model and heuristic method are detailed in Section 4. Numerical experiments, concluding remarks and future works are, respectively, given in Section 5 and Section 6.

## 2. The Drone Swarms Routing Problem

The Drone Swarms Routing Problem (DSRP) is defined on a complete and simple graph G=(V,E) built over a post-disaster area. Each search area is a vertex v∈V={0,1,⋯,n} which has an expected number of victims $e_{v}$ and a victim identification probability $p_{v}$. The set of edges is $E=\{\left(i,j\right)|i,j\in V^{2}\}$, where (i,j) is the shortest path between *i* and *j*. Moreover, each edge (i,j)∈E is weighted by a related cost $c_{ij}$ that represents the battery consumption of a single-drone flight starting from vertex *i* to *j*. Let D={1,2,⋯,m} be a homogeneous fleet of *m* drones, in which each drone d∈D has a range limit *A* according to its battery capacity, a battery recharge time *R* and an associated base $b_{d}\in B\subset V$ to perform the take-off, land and recharge operations. A specific drone searches for victims on a search location during a period of time $t_{s}$. The identification operation follows a binomial distribution evpv. As a consequence of the binomial distribution, search areas may be visited more than once and this visit can also be performed by the same drone. Another characteristic of the searches is that, at the same period of time, more than one drone (drone swarm) can scan for victims over the same search area. This latter characteristic is actually a relaxation of the problem that, on one hand, can improve the effectiveness of victim identification, but, on the other hand, it increases the search space of solutions to the problem, making the problem even more complex.

The DSRP considers a trajectory as a direct flight from a vertex *i* to another vertex *j*, consuming $c_{ij}$ of the battery. Trajectories can be performed by a drone swarm flying in a V-shaped formation with leader replacements. A “trip” in the DSRP is a circuit of a drone that departs from its base, flies to search areas, performs victim detection, and returns to the base. In the DSRP the whole operation of a drone may include multiple trips and there is a maximum time *T* allowed for this operation. The objective of the DSRP is to define routes for a fleet of drones in order to maximize the expected number of detected victims.

The DSRP involves some assumptions to facilitate the modeling and analysis of the problem. The first relies on the drone movements which are constrained to two dimensions, assuming a flat surface or environment. This simplifies the spatial representation and allows efficient route planning within a 2D space. The second assumption is the communication between drones which is always considered available. This second assumption enables information sharing and real-time coordination, which facilitates efficient swarm behavior and collaboration during routing tasks. On the other hand, a more realistic communication model could be taken into account as a constraint in our model. In summary, the focus is on the decision optimization problem, and this first approach does not consider speed and altitude variation. However, since the speed and altitude are constant, the solutions generated belong to the set of feasible ones.

**Figure 1:**
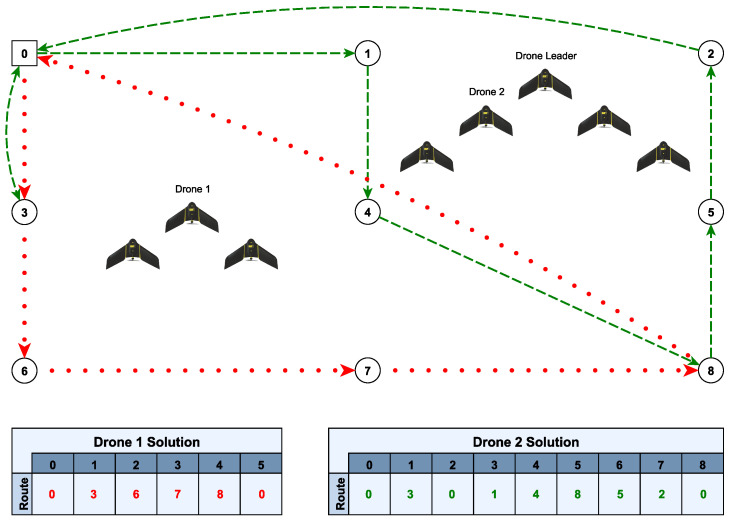
Example of a DSRP with eight drones, one base and eight search areas.

Figure 1 shows an example of a DSRP in a graph with nine vertices, eight drones and one base. It also contains two examples of routes performed by drones 1 and 2. Drone 1 starts the route by being the leader of the V-shaped formation, while drone 2, in turn, initiates the route by following another leader in another V-shaped formation. It can be seen that drone 2 performs two trips, as it returns to the base to be recharged.

Clearly, the DSRP is an NP-hard problem, as it originally comes from the Vehicle Routing Problem (VRP) [7], a well-known NP-hard problem. In this way, the time needed to find an optimal solution for the offline version of the DSRP can be impractical. Considering that the main focus here is to find a solution for the online version of the DSRP, the need for an efficient algorithm is even greater. This has motivated us to develop a heuristic to solve the DSRP problem.

## 3. Related Literature

This section provides a concise overview of closely related works in the literature, aiming to establish a comprehensive understanding of the research landscape. The review begins by examining optimization surveys that offer valuable insights into the current state-of-the-art techniques. Then, offline and centralized approaches are described for Drone Routing Problems, exploring different methodologies employed to optimize drone routes in the context of post-disaster management. In the following, online and decentralized Multi-Robot Task Allocation approaches are presented, showcasing an innovative strategy for real-time decision-making and coordination among drones. In addition, a study focusing on a V-shaped flight formation for drones is detailed, highlighting recent advances in flight formations that enhance efficiency and cooperation using drone swarms. The section ends with an overview comparing our contributions to those found in the scientific literature.

### 3.1. Optimization Surveys

In the scientific literature, there is growing interest in the use of drones, as can be noticed by the number of surveys published and the number of papers they analyze. From 2001 to 2017 more than 200 papers with optimization approaches for civil applications of drones were reviewed in [8], such as area coverage, search operations, routing, data gathering, networks of drones, communication linking and computing power to mobile devices. In [9], the authors classified 79 relevant publications from 2005 to 2019 that address Drone Routing Problems (DRP) modeled as a VRP. Survey [10] reviewed 63 papers, from 2015 to 2020, related to routing problems for four main problem classes: (1) the traveling salesman problem with drones; (2) the vehicle routing problem with drones; (3) the drone delivery problem and (4) the carrier-vehicle problem with drones. A survey and a framework for classifying drone-based delivery systems is presented in [11] and this framework was used to classify 101 related papers according to their objectives, methods, applications and constraints. The authors in [12] did an extensive analysis of articles published from January 2005 to June 2022, identifying 135 articles that explore various aspects of VRP problems. These surveys are very interesting entry points for researchers looking for the state-of-the-art on a wide range of topics involving optimization problems involving drones, like exact and heuristic solution methods, novel problem variants like drone routing, and emerging research areas such as green routing.

The surveys aforementioned surveys contain a very limited number of articles focusing on post-disaster management. This highlights a potential research gap in exploring the application of DRP methodologies to this constrained context. In addition, it is worth noting that a significant portion of the papers predominantly address the classic version of the VRP, which differs from the approach adopted in this work. This distinction emphasizes the contribution of this study in exploring novel perspectives and approaches that extend beyond traditional VRP formulations, aiming to address the specific challenges and complexities inherent in post-disaster management scenarios.

### 3.2. Offline and Centralized DRP Approaches

Some studies investigate variants of DRPs in the post-disaster context, proposing both methods and case studies. The DRPs can be addressed by adopting an offline or online approach. Offline computation is very often used for static and centralized approaches when there is enough time for decision-making. On the other hand, online computation is commonly used for dynamic and decentralized approaches, when the time for decision-making is a hard constraint, being taken in almost “real time”. All the works cited in this section consider offline computation with a centralized approach.

The authors in [13] address a DRP to recognize a post-earthquake area, where the objective is to find the shortest path to visit each site affected by the earthquake and to check the state of building damage. A simulated annealing was proposed and was applied to instances generated from the city of Acireale (Italy), finding good solutions with two drones. Another DRP to assess post-disaster areas using drones and motorcycles is studied in [14], where a bi-objective mathematical model for maximizing the total importance of population centers (nodes) and road segments (arcs) is presented. The bi-objectives are optimized by means of an ϵ-constraint method, together with a heuristic to solve the problem on instances based on Istanbul’s Kartal district (Turkey). The results show that a high-quality approximation of the Pareto front can be found using the proposed methodology.

Study [15] presents a DRP that uses gliders to collect useful information for assessing the extent of the disaster’s consequences by visiting the post-disaster locations as fast as possible. They also propose a solution to this problem by linearizing a Mixed-Integer Nonlinear Programming (MINLP) formulation that optimizes the routes and the trajectories along these routes. The MINLP formulation is tested on random instances and on instances based on flooding-prone cities of the United Kingdom (UK). These instances are divided into small and medium ranges and they contain up to 10 waypoints, 2 landing zones and 3 gliders. The strategy is able to prove optimal solutions for some test cases, contrary to large test cases with more waypoints and a medium to large size. A heterogeneous fixed fleet DRP is considered in [16] with the objective of minimizing the inspection cost of a post-disaster area. In order to solve it, the authors propose a Mixed-Integer Linear Programming (MILP) model and two heuristics, an Adaptive Large Neighborhood Search (ALNS) algorithm and a Modified Backtracking Adaptive Threshold Accepting (MBATA) one. The algorithms were tested on instances generated over Hancock county from Mississippi State (USA) and produced high-quality solutions in a reasonable time.

### 3.3. Online and Decentralized MRTA Approaches

Considering the field of multi-agent systems (MAS), the Multi-Robot Task Allocation (MRTA) is a problem commonly found problem (see [17] for more information on MRTA). The MRTA is an analogous problem to the VRP in which tasks are defined in terms of location. Furthermore, the MRTA problems can be also solved by adopting offline or online approaches.

Several studies consider MAS with an online approach to solve different MRTA problems, but there is a lack of studies that address MRTA in post-disaster areas. The authors in [18] present a MRTA in which robots (drones) with limited range and payload must accomplish tasks with deadlines that are generated during the search. The MRTA goal is to deliver survival kits to spatially distributed victims after a flood disaster. An Integer Linear Programming (ILP) formulation is presented to solve the offline version of the problem as well as an online method, named BiG-MRTA, that combines a bipartite graph construction with a model that assigns edge weights to the graph, and allocates tasks, by solving a maximum weighted matching problem. Each task can be allocated to only one agent and task selection is performed in a myopic way, i.e., at each iteration, an agent selects the next task to undertake considering an acquisition function that balances exploitation and exploration. The algorithms were run on instances generated from South Hilo district, Hawaii. The results show that the BiG-MRTA is more than $10^{3}$ times efficient than the ILP, and it can also offer up to 46% higher task completion when compared to a random walk baseline in problems with 1000 tasks.

Some studies in the literature investigate several dynamic issues (velocity, communication delays, etc.) of coordinating drones swarms, as for instance in [19,20].

### 3.4. V-Shaped Formation

Another inspiration for our work comes from [3], in which the authors study the energy conservation of V-shaped swarming flight for fixed-wing drones. The study has shown that the V-shaped formation drones can save up to 70% of their energy, and that adopting leader replacements during the flight, a further 21% of their energy is saved, consequently, increasing the flight time and the distance travelled.

### 3.5. Position of This Study

Table 1 summarizes the set of closely related studies and compares them with this study in terms of the problem addressed, the objective function, the proposed method (s) and the case study.

Analyzing Table 1, the lack of works that study DRPs using multi-agent systems in a decentralized approach and performing online computation is clearly noticeable. Moreover, no other work models a DRP that considers cooperation between drones that can fly in V-shaped formations with leader replacements. Another point to be highlighted is related to the objective addressed since the search for the maximum number of victims in a post-disaster context is considered crucial for disaster management.

## 4. DSRP Computational Model and Methods

In the DSRP multi-agent decentralized approach, each drone is modeled as an agent able to compute its route based on real-time information. The drones can decide when it is appropriate (or not) to work cooperatively in a swarm of drones, considering a V-shaped formation with leader replacements. This decision is addressed by means of a heuristic proposed here. It should be noted that two versions of this model have been developed. The first is the offline version, whose main feature is the fact that when a drone searches for victims at a search location, the number of victims identified will always be equal to the expected number. As for the other version, the online version, the number of victims identified will be obtained at the instant of identification, and may be greater or less than the expected number.

**Figure 2:**
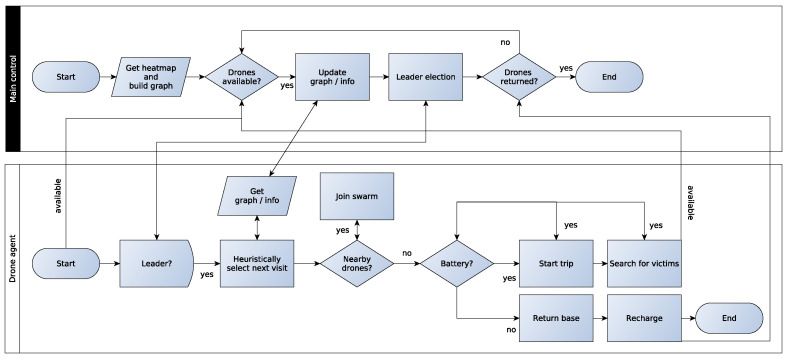
Flowchart of the computational model for the DSRP.

Figure 2 shows a flowchart of the proposed decentralized model. This algorithm contains two main modules: the Main Control (MC) and the Drone Agent (DA). The MC is responsible for building the heatmap of the post-disaster area containing all the information about possible victim locations. In the sequel, a graph based on this heatmap is built. Then, the algorithm checks which drones are available, updates the graph by getting their new information and sends it to the available drones. In the following, the MC performs a leader election to choose a leader. Finally, the MC controls the return of the drones to the base, considering the start of a new operation or the end of the whole operation.

The DA starts its work by becoming available and communicating with the MC. Thus, it waits for the leader election and when the answer from MC is positive the Drone Swarm Heuristic algorithm is executed to choose the next node to visit in the next trip. This decision is made by obtaining the updated graph and information about the other operations from the MC. In this module, it also decides about the drone swarms’ V-shaped formation, by making the DA able to join a nearby drone swarm, and the leader replacements during the flight. In order to perform any operation, the DA must check whether it will have enough battery power to reach the next node, search for victims and return to the base. Thus, the DA performs its operation, which may be to start the trip to the next node and then search for victims at this node; otherwise, it stops and returns to the base station. Finally, after finishing its operation, the DA reports to the MC if it will be available for the next operation or if it has finished all its work.

By employing an auction-based algorithm, for example, the Consensus-Based Bundle Algorithm (CBBA) [21], decentralized coordination can be achieved, enabling agents to effectively exchange information between themselves. Such an approach fosters distributed decision-making, increased autonomy and robustness in the face of uncertainties or disruptions. With regard to the architecture of the data communication network, we can cite some recent works that address interesting architectures in which our model can be easily incorporated, making it possible to deploy it in real disasters. In [22] we are introduced to a communication network architecture for a squadron of drones to be used in the scanning rocket impact area of Barreira do Inferno Launch Center in Rio Grande do Norte, Brazil to detect intruder boats. The system uses Wireless Sensor Networks, specifically employing XBee Pro 900HP S3B modules, for information transmission within a range of 5 km.

The remainder of this section describes in more detail both modules of the algorithm, Main Control (Section 4.1) and Drone Agent (Section 4.2). It is important to note that both modules contain simple and reproducible algorithms.

### 4.1. Main Control

The main control module contains five sub-modules: Get heatmap and Build a graph, Drones available, Update graph/info, Leader election and Drones returned. These sub-modules are detailed next.

**Figure 3:**
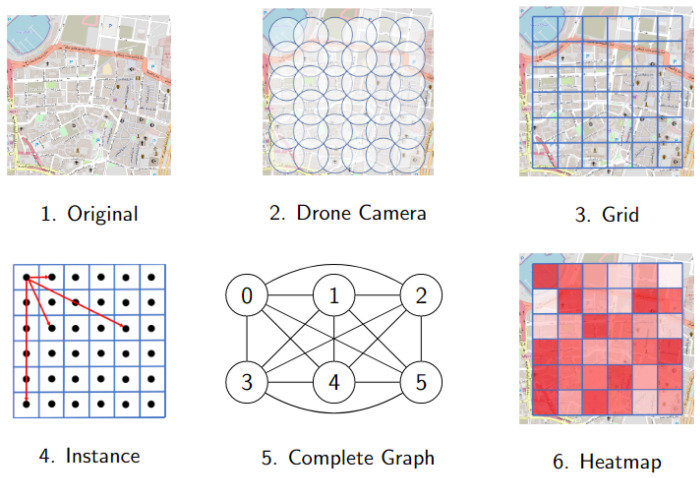
How to build the heatmap and transform it into a complete graph.

*Get heatmap and build graph*. The heatmap is essential information to start the search for a solution to the DSRP. The procedure on how to build the heatmap and transform it into a complete graph is illustrated in Figure 3. This procedure consists of five steps.

The first step consists of obtaining the map of the post-disaster search area through some data source, for instance, the open data source OpenStreetMap (OSM). Then, the second step consists of defining all possible search locations for the drones, and setting the radius of action according to the range detection of the drones. The goals are to identify victims and cover the entire search area using this information.

The third step looks for the data related to the expected number of victims ($e_{v}$) and the victim identification probability ($p_{v}$) in each possible search location. Such information can be obtained from official government data, as well as through unofficial data from other sources (ground teams, Non-governmental organizations, etc.) The $e_{v}$ number can be deduced, for example, from the number of buildings existing before the disaster. The probability $p_{v}$ is defined by taking into account the difficulty of identifying victims in each search area after the disaster has occurred. The more buildings there are, the higher the expected number of victims, and the lower the probability of identifying them. Figure 2 shows each search area colored in tints of red, in which the deeper the tint, the higher the estimation of the number of victims.

In the fourth step, the conversion of the heatmap into a complete graph is initiated, by first building a grid graph. This transformation is made as follows. Each search area of the grid represents a vertex of the graph, and the edges connect adjacent search areas. The edges are weighted by the battery consumption related to the path of a single drone. Once again, the number of buildings can help to define these weights.

In the last step, the complete graph is built, by solving the All Pairs Shortest Path Problem and then connecting all pairs of vertices with edges weighted by the minimum battery consumption between them, i.e., the one that corresponds to the shortest path between such vertices in the previous graph.

This step allows the drone availability to be checked. At this stage, there are three possibilities for receiving messages concerning the availability of drones. Two of them are treated in trivial ways, i.e., as soon as the message is received the process continues its flow directly to the next step. The third possibility requires synchronization as well as consistency of the graph information. The two trivial possibilities are when the DA module starts its processing and after the drone finishes its battery recharge. In the latter case, the message is only sent if there is still enough time to visit at least one search location. The last one is when the drone just ends up performing a search for victims operation. In order to avoid exchanging too many messages with the MC module, it is considered that at the end of the search, the current leader will control the availability of his swarm and he will be responsible for sending the message to the MC, notifying the swarm availability and also containing new information about the number of victims identified by the swarm.

*Update graph/info*. In the Update graph/info step, the information obtained by the leaders regarding the number of victims identified by the drones is updated in the graph. In addition, information related to the current allocation of drones to search locations is also updated. Considering this information, it is important to limit the number of drones that are on their way to the same search location.

*Leader election*. This step performs the choice of an available drone to be the next leader. This choice is performed according to a very simple rule: the next leader will be the one with the largest amount of available battery. In case of a tie, the next leader will be chosen randomly among those with sufficient battery.

*Drones returned*. Drones returned is the last sub-module of MC and is responsible for controlling the return of drones to the base. Basically, it is performed as a check on the remaining time of the whole operation, considering the maximum time available *T*. If there is still enough time for the drone to visit at least one search location and still return to the base, then the drone becomes available and this message is transmitted to the DA sub-module. Otherwise, the DA module of such a drone is concluded. In this way, the MC must wait until all the drones have no more time left for the operation to stop the algorithm.

### 4.2. Drone Agent

As mentioned before, the DA module starts by sending a message indicating its availability to the MC. Then, the processing continues following the flow of its eight sub-modules detailed next: Leader, Get graph/info, Drone swarm heuristic, Join swarm, Battery check, Start trip/Return base, Search for victims and Recharge.

*Leader*. In this step, the agent waits for the leader’s election. Once it is defined, the agent immediately moves on to the next step, the heuristic choice based on updated information.

*Get graph/info*. Before choosing the next search location to visit, the agent updates the information regarding the graph, as well as the operations that other agents are performing at the moment. These data are essential to make the best decisions considering the current configuration of the environment. Hence, the DA requests, and obtains, from the MC the current information which, more specifically, corresponds not only to the expected number of victims remaining at each search location but also to what are the trips that other agents are performing at that moment.

*Drone swarm heuristic*. The Drone Swarm Heuristic (DSH) will build iteratively a complete solution for the DSRP based on two criteria: the expected number of victims and operation cost. The choice to define only the next visit at each moment is necessary to guarantee the applicability of the model. Thus, the drones make this decision promptly, avoiding a bottleneck in the model and consuming less energy. Initially, DSH uses the data obtained from the previous sub-module to know the possible search locations available to be visited by the agent, this information is referred to here as the candidate list (CL). It means that if a search location has a positive remaining number of expected victims, then it will be included in the CL. Otherwise, if this number is close to zero, it means that all expected victims were identified. Then, DSH will rank each search location *v* according to the F function (1):

(1) $$F\left(i,v\right)=\frac{e_{v}\times p_{v}}{c_{ij}\times swarm\left(v\right)+t_{s}},$$

which is responsible for obtaining, given a start location *i* and a search location *v*, the ratio of the expected number of remaining victims ($e_{v}\times p_{v}$) over the operation cost (the trip cost $c_{iv}$ may be reduced according to the number of drones in the swarm). The objective is to maximize *g* in CL, the $F_{max}=\max\left\{F\left(i,v\right)\;|\;\forall v\in CL\right\}$. In other words, the $g_{max}$ represents the search location that provides the highest victim identification while also taking into account the cost of this operation.

It is noteworthy that the DA can only choose feasible search locations to visit, taking into account enough battery to fly to the location, detect and return to the base station.

*Join swarm*. Once the decision on the node to be visited is made, the agent has the possibility of joining up with another agent or a swarm that is making a common trip. Even though they may have different final destinations, agents can group together to make an intermediate trip and split up afterward.

In [3], the authors presented some possible scenarios of savings when using a drone swarm performing flights in a V-shaped formation. Among all of them, in this work, we adopted the one that the authors consider the closest to real applications, i.e., reducing to 10%, 15%, 19% and 21% of energy consumption by using two, three, four and five drones. In addition, in [3] the possibility of reducing to 20% of extra savings by adopting the V-shaped formation with leader replacements in each case is also presented. Table 2 presents the savings of this realistic scenario, considering the V-shaped formation and leader replacements according to the number of drones. In the last row (Total savings) we present the combined savings adopted by our computational model. To clarify, in our model, if the energy consumption between two locations is 100 and there is a swarm of two drones flying between them, this means that we will have a final consumption of 72 (28%) because of the reduction in total consumption, being 90 (10%) because of the flight in a V-shaped formation and finally 72 (20% extra) because of the replacement of leaders. It is noticeable from the table that drone swarms are limited to a maximum of five drones. This means that during the whole operation at most five drones are allowed to be in the same V-shaped formation. If more than five drones fly to the same search location, then the model will allow at least two different swarms to coexist.

The swarm function used by Equation (1) works as follows. According to a search location *v*, the swarm function returns the corresponding saving value related to the number of drones that visit *v*.

*Battery check*. The agent must check its remaining battery during the whole operation to prevent the drone from dropping during its flight. The Battery check sub-module periodically performs such a check, considering that the agent must always have enough battery to return to its base. If, for any reason, the remaining battery is not enough to continue the current operation, this sub-module triggers the immediate return of the agent to the base.

*Start trip/return base*. The Start trip/return base sub-module is responsible for conducting the trip for the agent. An agent can perform the trip by itself or in a swarm of drones, in the latter case the trip cost will be lower for all drones involved in this cooperation. When the agent is running out of battery, it will perform a return to base, which is also conducted as a normal trip, alone or in a cooperative flight.

*Search for victims*. The search for victims is an action of an agent that demands a fixed time. If this action is carried out by more than one drone the time required will be similar for each. However, the more agents there are, the greater the probability of identifying victims. At the end of this sub-module, the DA leader sends a message to the Drones available sub-module reporting not only on the number of victims identified but also on drone availability for future operations.

*Recharge*. The recharge of an agent requires a fixed time, which must be respected until the end for the recharge to take place, i.e., it is not possible to recharge an agent partially. Once the recharge is complete, the DA notifies the Drones returned sub-module of the MC that the battery is full and whether there is still enough time to perform any further operations. If there is not enough time, in addition to notifying the MC, the agent terminates its activity.

## 5. Computational Experiments

All the algorithms were implemented in C++, using the Windows/GNU g++ compiler (version 8.1.0). All tests with the proposed algorithms were executed on an Intel(R) Core(TM) i5-9300H @ 2.40 GHz processor with 16 GB RAM on Windows 10.

The solutions produced by the proposed methods were adapted to be visualized using the simulator CoppeliaSim [6]. This allows the execution of the solutions to be followed dynamically.

The goals of the numerical experiments are to check the performance and the impact of the proposed offline and online methods using the realistic case study. For such a purpose, several indicators are used such as:

- *O*→ total number of operations achieved by all drones;
- $O_{c}$→ the number of operations achieved in cooperation;
- *T*→ the total number of trips;
- $T_{v}$→ the total number of trips in a V-shaped formation;
- *S*→ the total number of scans;
- $S_{c}$→ the total number of scans in cooperation;
- *R*→ the total number of recharges;
- end→ the time the last drone returns to base;
- avg→ the average time the drones return to base;
- *C*→ the total cost in time units to conclude all operations: to take off from the base, arrive at search locations, identify victims, return to a base and recharge;
- $\bar{C}$→ the total cost in time units to conclude all operations without considering savings from the V-shaped formation flight;
- G(%)→ the percentage gain obtained by considering the savings;
- *E*→ the total number of expected victims;
- *V*→ the total number of victims identified by the drones;
- V(%)→ the percentage related to the number of victims found.

In the following sections, the case study, the development and settings and results considering both offline and online approaches, together with sensitivity analysis are detailed in the next sections.

### 5.1. Case Study: Beirut Port Explosion

**Figure 4:**
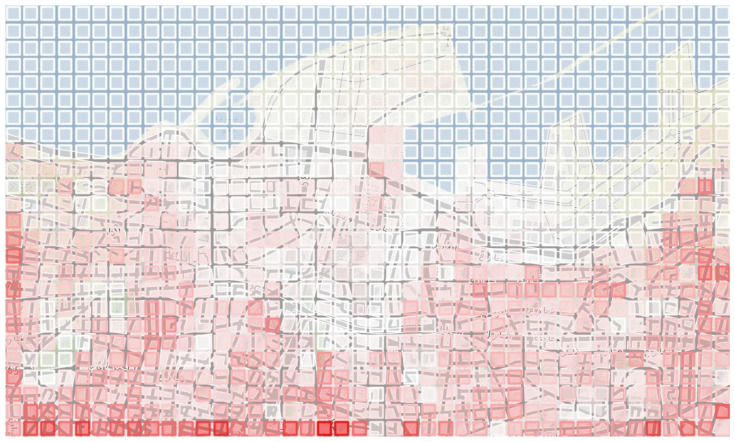
Case study: Beirut Port explosion.

The Beirut Port scenarios were generated with the instance generator coming from the study [5], using additional features for the DSRP. The process was described in Section 4.1. A fleet of drones able to communicate with each other is defined. The complete graph is obtained by All Pairs Shortest Path Problem. Figure 4 shows the selected area divided into squares of 100 m${}^{2}$, colored in tints of red to illustrate the expected number of victims. The dimension of the grid graph constructed is 42 × 25, thus resulting in a complete graph of 1050 vertices.

#### Scenarios

Three scenarios, referred here as Scenario 1, 2 and 3, were generated with 1050 vertices, 2 recharge base stations, and 8 drones. Each drone has a range limit of 3540, a recharging time of 2400, and the time spent detecting victims equals one. These values are based on the eBee senseFly drone [23], which was also set and used by [3]. The maximum time for the whole operation is 7200. The differences between the three scenarios are the variation in the expected number of victims in each search area (totaling 29,349, 30,599, and 31,711, respectively). They also differ in the identification probability for each search area.

### 5.2. Development and Settings

The MC module makes use of a main thread, while the DA uses more than one thread, working in parallel. This distinguishes our development from that of [18], who employ only one thread.

**Figure 5:**
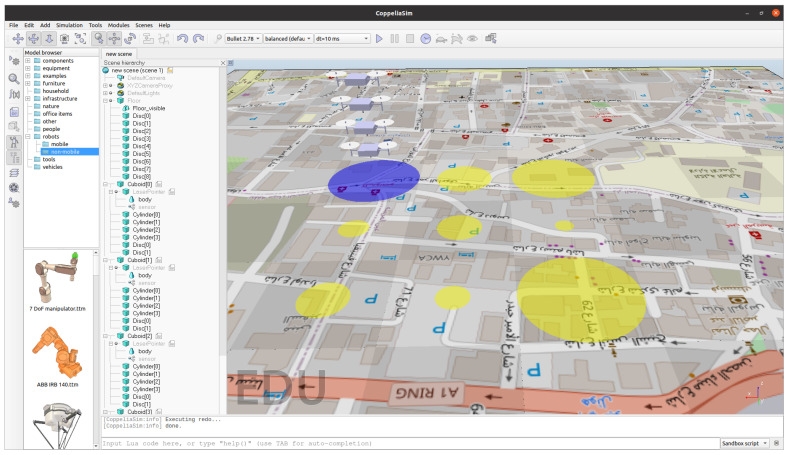
Example of a solution loaded in CoppeliaSim.

Figure 5 shows the interface of CoppeliaSim as well as an example of a loaded solution for the DSRP, containing four drones and nine vertices (one base and eight search areas). Each drone is positioned at a certain height in order to facilitate viewing, even if they are in a V-shaped formation. This has been conducted to simplify the simulation. A code color is used to identify the leaders, the drones following the leader, drone recharging, for example, the yellow and blue vertices that represent, respectively, the base stations, and the potential nodes with victims. The size of each yellow circle is proportional to the expected number of victims in the corresponding area.

### 5.3. Results Using an Offline Approach

The performance of the proposed algorithm is also evaluated in an offline approach based on the Beirut Port explosion. In the offline version, the number of victims obtained in each search location will always be equal to the expected number. For instance, if it is expected that there are 100 victims in a search location and the identification probability is 60%, then for each victim search operation, 60% of the remaining victims will be identified, i.e., 60, 24, 9.6, 3.84, etc.

#### 5.3.1. Sensitivity Analysis on the Number of Drones

In this experiment, the number of drones varies (4, 8, 16, 32 and 64), while the other parameters remain similar along the optimization. Table 3, Table 4 and Table 5 present results using the aforementioned indicators.

Obviously, the more the number of drones increases, the higher the percentage of identified expected victims is. It is noteworthy that with only four drones it is possible to reach more than 91% of identified victims. When increasing to eight drones, this percentage reaches more than 99% of the expected victims. Finally, with 16, 32 and 64 drones, practically all victims are expected to be identified. Taking into account the modest savings model applied, it is considered that reasonable gains can be achieved through the use of V-shaped formation flight, reaching values above 3%.

**Figure 6:**
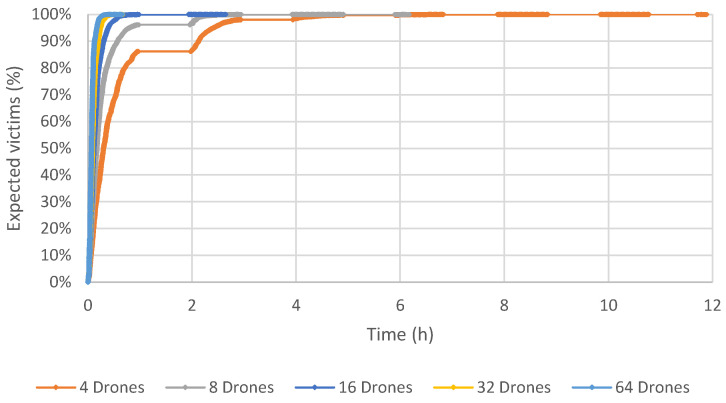
Scenario 1: Results varying the number of drones, in terms of time and percentage of expected victims.

**Figure 7:**
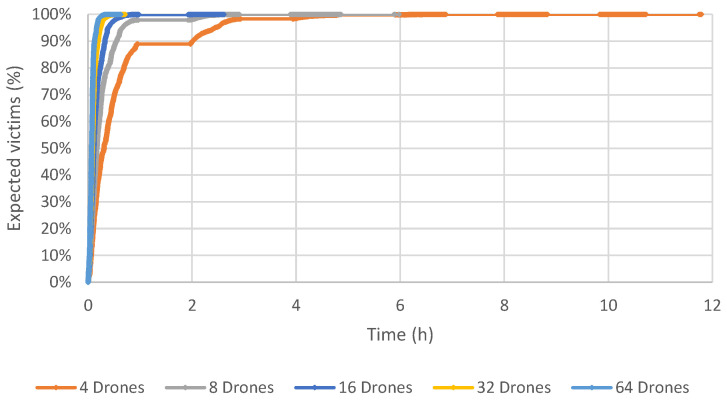
Scenario 2: Results varying the number of drones, in terms of time and percentage of expected victims.

**Figure 8:**
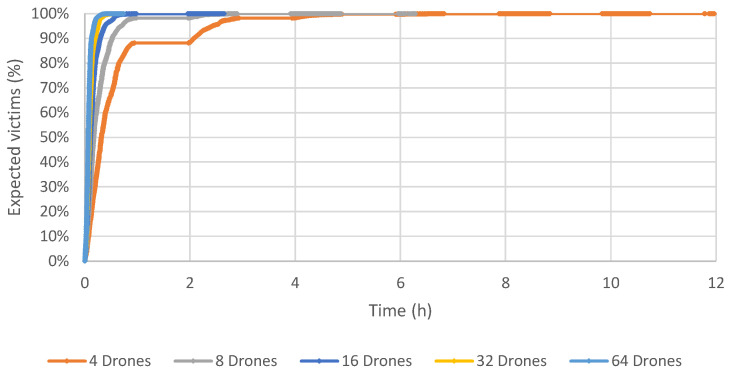
Scenario 3: Results varying the number of drones, in terms of time and percentage of expected victims.

Results using 32 and 64 drones show that the final time spent concluding all operations is less than the maximum time allowed. Due to that, the maximum time limit used in the simulation was removed, in order to check the impact of using each number of drones. Figure 6, Figure 7 and Figure 8 depict a graph that contains the percentage of expected victims identified by our computational model over time and according to the number of drones for Scenarios 1, 2 and 3.

These results show that the proposed method obtains a good coverage of the post-disaster area of Beirut in a very acceptable time. Using 64 drones, the time required to reach all expected victims is about 40 min. If the number of drones equals 32, this time increases to one hour. All the expected victims were found using 16 drones and about 2.5 h. Six hours are necessary to identify all the expected victims using eight drones. Not surprisingly, the longest time to identify all the victims is when there are only four drones, taking slightly less than 12 h.

#### 5.3.2. Sensitivity Analysis of the V-Shaped Formation with Leader Replacements

**Figure 9:**
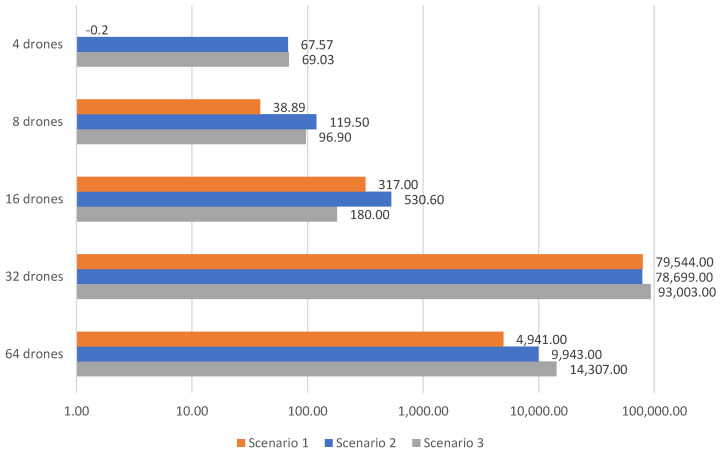
Additional cost required without V-shaped formation savings.

**Figure 10:**
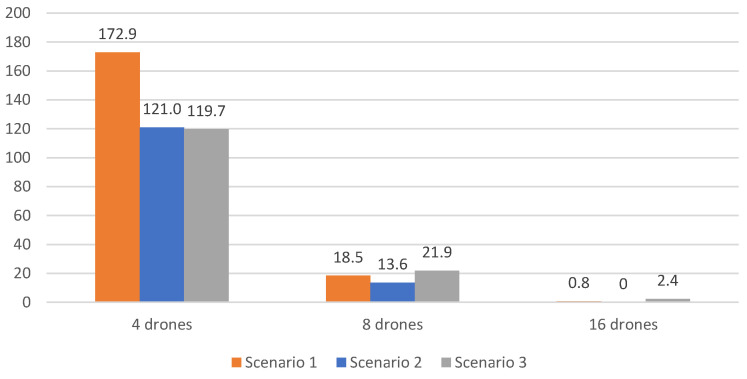
Number of extra expected victims identified with V-shaped formation savings.

Table 6, Table 7 and Table 8 present the results obtained without using a V-shaped formation with leader replacements, respectively, for Scenario 1, 2 and 3, using 4, 8, 16, 32 and 64 drones. Moreover, Figure 9 and Figure 10 consider, respectively, the additional cost and number of victims found, whenever not considering the savings related to the V-shaped formation flight with leader replacements.

Results clearly indicate that not using V-shaped formations with leader replacements led to a smaller number of victim detection and a higher cost, compared to the previous results. The cost is increased in up to 80%, and the victim detection is decreased up to 1% It is worth noting that the number of recharges also increases for 32 and 64 drones.

Figure 9 illustrates higher cost, except with four drones in scenario 1, which was slightly lower, −0.20. Using more drones, 32 and 64 drones results in a higher cost, especially using a V-shaped formation. This is due to the additional recharges required. On the contrary, the number of victims identified increases, as depicted in Figure 10. Using 32 and 64 all victims are identified, while the improvement using fewer drones (4, 8 and 16) is noticeable. On average, 137 extra victims could be identified with 4 drones, 18 extra victims with 8 drones and 1 extra victim with 16 drones. Such results demonstrate the importance of adopting the savings related to the V-shaped formation strategy.

#### 5.3.3. Sensitivity Analysis on the Detection Probability

In these experiments, we evaluated the impact of using a detection probability associated with the search locations. For this purpose, three levels of detection probabilities, defined by means of intervals, were used: low ([0%,33%]), medium ([33%,66%]), and high ([66%,100%]). Values were randomly generated within these ranges for each search location of each scenario. The results obtained for scenarios 1, 2 and 3 using eight drones are shown, respectively, in Table 9, Table 10 and Table 11.

It is remarkable for the three Scenarios, that even with low identification probabilities, the proposed computational model was able to find a high percentage of expected victims, more than 87%. Another interesting observation is that in these more "adverse" situations, the method makes use of more flights in a V-shaped formation. This can be seen when observing the highest gains in savings and, of course, the number of squad operations. Whenever the probabilities were applied at the medium level, more than 98.7% of victims were identified in all Scenarios, obtaining savings of more than 1.3% with V-shaped formation flight. By using the level with a high identification probability, the percentage of identified victims was greater than 99.6% and the savings gain was about 1%. This happens since the use of V-shaped formation flight is not as necessary as in the low detection probability level.

#### 5.3.4. Sensitivity Analysis on the Number of Expected Victims

The goal of these numerical experiments is to evaluate the impact of the method when the expected number of victims at each search location is underestimated or overestimated. Therefore, the Scenarios were adapted to this case. In an underestimated case, each search location has the number of victims decreased to a random value between 50% to 75%, 25% to 50%, and 0% to 25%, considering the initial number of victims. In the overestimated case, the number of victims was increased in each location as a random value between 0% to 25%, 25% to 50% and 50% to 75%, considering the initial number of victims. Results are depicted in Table 12, Table 13 and Table 14, using eight drones. The previous results obtained in Table 3, Table 4 and Table 5 are presented in line “Initial Scenario” to allow comparison.

Results remain stable for underestimated and overestimated cases, i.e., more than 99% of the expected victims are found, with gains in savings with flights in V-shaped formation between 1% and 2%. These results allow the robustness of the proposed method to be evaluated and, as a consequence, its applicability in real situations.

### 5.4. Results Using an Online Approach

In post-disasters, the probability of detecting victims in a search location may vary in every scanning situation. Thus, at each location, the number of victims identified is not always equal to the expected number. To address this context, an online version of the proposed method is tested, using the underestimate and overestimate cases, considering the variation of the detection probability. Both have the following intervals: [0–5%], [5–10%], [10–15%] and [15–20%]. For instance, whenever applying an underestimated case related to the interval [0–5%] over a search location where identification probability equals 60%, the underestimated case varies from 55% to 60%.

Table 15, Table 16 and Table 17 present the results obtained for the online approach, using the three Scenarios 1, 2, and 3. The proposed method seems robust for the Scenarios used, even in the most adverse situationwhere the probabilities decrease between <15% and 20%, the model was able to locate more than 97% of victims. Applying the overestimated case and the interval >15% and 20%, the method is able to identify almost all the victims. In summary, for these Scenarios, the approach deals very well with uncertainties related to the identifications.

## 6. Conclusions

In this article, the Drones Swarm Routing Problem (DSRP) is proposed, motivated by the application of finding victims after a disaster. The main novelty of the DSRP is to address a cooperation issue between drones. Both online and offline strategies are tested using drone cooperation. This is conducted with multiple drones and using a V-shaped formation with leader replacements that lead to less energy consumption.

A computational model that applies the Drone Swarm Heuristic and uses multi-agents in a decentralized approach is proposed. It has decision features allowing a drone to operate alone or in a swarm. It relies on the hypothesis that drones are able to correctly fly within a group without collisions, considering both path planning and detection. This assumption is used since we are focusing on the decision optimization problem. The proposed computational model was developed using threads. Solutions obtained were also tested using the robot simulator CoppeliaSim, in order to visualize the operations of drones over time.

A case study was conducted based on the Beirut port explosion, thereby generating three test Scenarios. They were tested using an online and an offline approach. Extensive numerical experiments were run for the off-line version: varying the number of drones; enabling and disabling a flight-related V-shaped formation with leader replacements; varying the identification probability, and the number of expected victims. In the sequel, the online version was studied, where the identification probability was randomly modified along the test.

For the Scenarios used in this study, the proposed heuristic methods are robust. Moreover, using eight drones, results indicate that more than 99% of victims can be found within 2 h. Using 16, 32 and 64 drones, a slight improvement is obtained, despite a higher running time. The V-shaped formation with leader replacements gives very interesting results, making it possible to find extra numbers of victims at a lower cost. In general, the proposed heuristic method has good coverage.

This study opens several avenues of research. The communication issue, which in practice consumes a large part of the energy consumption can be investigated together with other types of topologies for drones, for example, the work of [24] presents an architecture including aerial base stations that must provide network services to ground users who can move around. Other methods, such as 3D search space with Reinforcement Learning [25] or even artificial potential fields [26], can be investigated in order to carry out the path planning and also guarantee the permanence of the V-shaped formation. In addition, the detection is not explicitly integrated in this study. In fact, it is possible to accomplish that using thermal cameras, or even artificial intelligence using images of the areas. This step is an area for forthcoming study. Moreover, it is noticeable that observations at a higher altitude allow larger areas to be scanned at once, in spite of lower precision. On the contrary, a lower altitude allows more local and precise observations. Analyzing the trade-off between higher and lower altitudes is left for future work.

## Figures and Tables

**Table 1:** Comparison of closely related works with this study.

Authors	Problem Addressed	Objective Function	Method(s)	Case Study
**Cannioto et al. (2017) [13]**	Drone routing	Minimize travel distance	Metaheuristic, centralized	Sicily, Italy
**Oruc and Kara (2018) [14]**	Drone routing	Maximize assessed road segments and points of interest	MILP, exact, heuristic, centralized	Kartal, Turkey
**Coutinho et al. (2019) [15]**	Drone routing	Minimize total flight time	MINLP, centralized	Cities, UK
**Chowdhury et al. (2021) [16]**	Drone routing	Minimize inspection cost	MILP, metaheuristics, centralized	Mississippi, USA
**Ghassemi and** **Chowdhury (2022) [18]**	Online Task allocation (Drone routing)	Maximize completed tasks	MILP, metaheuristics, decentralized, multi-agent systems	South Hilo district, Hawaii
**Our work**	Online Drone routing	Maximize identification of victims	Heuristic, decentralized, multi-agent systems	Beirut, Lebanon

**Table 2:** Possible savings for the drone swarms obtained from [3].

Number of Drones	1	2	3	4	5
**V-shaped formation**	0%	10%	15%	19%	21%
**Leader replacements**	0%	20%	20%	20%	20%
**Total savings**	0%	28%	32%	35.2%	36.8%

**Table 3:** Results produced using Scenario 1.

Scenario 1	*O*	$O_{c}$	*T*	$T_{v}$	*S*	$S_{c}$	*R*	end	avg	*C*	$\bar{C}$	*G*(%)	*E*	*V*	*V*(%)
**4 drones**	3249	17	1215	15	2030	0	4	7199.93	7199.48	28,797.9	28,903.1	0.37%	29,349	26,946.1	91.81%
**8 drones**	6592	300	2317	74	4267	221	8	7199.71	7194.58	57,556.6	58,383	1.44%	29,349	29,200	99.49%
**16 drones**	12,456	498	4757	215	7683	268	16	7199.14	7179.07	114,865	116,523	1.44%	29,349	29,347.4	99.99%
**32 drones**	14,898	1135	6257	404	8641	721	0	3370.75	3175.09	101,603	104,176	2.53%	29,349	29,348.5	99.99%
**64 drones**	17,797	1879	9976	1018	7821	829	0	2715.88	2461.86	157,559	162,719	3.27%	29,349	29,348.6	99.99%

**Table 4:** Results produced using Scenario 2.

Scenario 2	*O*	$O_{c}$	*T*	$T_{v}$	*S*	$S_{c}$	*R*	end	avg	*C*	$\bar{C}$	*G*(%)	*E*	*V*	*V*(%)
**4 drones**	3283	98	1163	25	2116	70	4	7199.97	7182.97	28,731.9	28,929.1	0.69%	30,599	28,970.2	94.68%
**8 drones**	6167	127	2380	59	3779	61	8	7199.74	7184.47	57,475.8	58,147.7	1.17%	30,599	30,439.6	99.48%
**16 drones**	11,502	416	4831	206	6655	128	16	7196.72	7164.71	114,635.4	116,536.6	1.66%	30,599	30,597.1	99.99%
**32 drones**	14,212	607	7108	273	7104	190	0	3533.23	3425.93	109,630	111,724	1.91%	30,599	30,598.5	99.99%
**64 drones**	16,651	968	9662	798	6989	155	0	2696.05	2343.24	149,967	153,857	2.59%	30,599	30,598.6	99.99%

**Table 5:** Results produced using Scenario 3.

Scenario 3	*O*	$O_{c}$	*T*	$T_{v}$	*S*	$S_{c}$	*R*	end	avg	*C*	$\bar{C}$	*G*(%)	*E*	*V*	*V*(%)
**4 drones**	3288	22	1197	16	2087	3	4	7199.82	7182.09	28,728.3	28,865.2	0.48%	31,711	29,522.8	93.10%
**8 drones**	6634	115	2378	87	4248	20	8	7190.95	7187.29	57,498.4	58,299.2	1.39%	31,711	31,544.3	99.47%
**16 drones**	12,902	279	4785	198	8101	65	16	7199.55	7186.52	114,984	116,448	1.27%	31,711	31,709.7	99.99%
**32 drones**	14,788	977	6585	408	8198	561	5	5688.02	3629.65	116,149	118,494	2.02%	31,711	31,710.5	99.99%
**64 drones**	18,340	1662	10,671	1137	7669	509	0	3188.75	2617.07	167,493	173,765	3.74%	31,711	31,710.6	99.99%

**Table 6:** Results for Scenario 1 without using V-shaped formation with leader replacements.

Scenario 1	*O*	*T*	*S*	*R*	end	avg	*C*	*E*	*V*	*V*(%)
**4 drones**	3133	1200	1929	4	7199.79	7199.42	28,797.7	29,349	26,773.2	91.22%
**8 drones**	6745	2300	4437	8	7199.96	7199.44	57,595.5	29,349	29,181.5	99.43%
**16 drones**	11,993	4519	7458	16	7199.91	7198.85	115,182	29,349	29,346.6	99.99%
**32 drones**	15,365	6575	8760	30	6587.26	5660.85	181,147	29,349	29,348.5	99.99%
**64 drones**	17,763	9498	8263	2	4561.89	2539.06	162,500	29,349	29,348.6	99.99%

**Table 7:** Results for Scenario 2 without using V-shaped formation with leader replacements.

Scenario 2	*O*	*T*	*S*	*R*	end	avg	*C*	*E*	*V*	*V*(%)
**4 drones**	3328	1185	2139	4	7199.99	7199.86	28,799.5	30,599	28,849.2	94.28%
**8 drones**	6153	2365	3780	8	7199.88	7199.41	57,595.3	30,599	30,426	99.43%
**16 drones**	11,472	4681	6775	16	7199.93	7197.88	115,166	30,599	30,597.1	99.99%
**32 drones**	14,305	7116	7157	32	6317.9	5885.28	188,329	30,599	30,598.5	99.99%
**64 drones**	16,738	9950	6788	0	2849.22	2498.59	159,910	30,599	30,598.6	99.99%

**Table 8:** Results for Scenario 3 without using V-shaped formation with leader replacements.

Scenario 3	*O*	*T*	*S*	*R*	end	avg	*C*	*E*	*V*	*V*(%)
**4 drones**	3568	1150	2414	4	7199.87	7199.33	28,797.3	31,711	29,403.1	92.72%
**8 drones**	6522	2333	4181	8	7199.81	7199.41	57,595.3	31,711	31,522.4	99.41%
**16 drones**	11,879	4660	7203	16	7199.97	7197.74	115,164	31,711	31,707.3	99.99%
**32 drones**	15,839	8021	7786	32	7098.45	6535.99	209,152	31,711	31,710.5	99.99%
**64 drones**	18,963	10,512	8446	5	4830.35	2840.62	181,800	31,711	31,710.6	99.99%

**Table 9:** Impact of using different levels of probability for Scenario 1.

Scenario 1	*O*	$O_{c}$	*T*	$T_{v}$	*S*	$S_{c}$	*R*	end	avg	*C*	$\bar{C}$	*G*(%)	*E*	*V*	*V*(%)
[0%,33%]	15,137	1687	1463	240	13666	1441	8	7199.94	7178.09	57,424.7	59,471.9	3.57%	29,349	25,636.7	87.35%
[33%,66%]	8401	167	2169	123	6224	38	8	7199.97	7162.62	57,301	58,372.5	1.87%	29,349	28,973.4	98.72%
[66%,100%]	5595	63	2464	56	3123	1	8	7199.73	7184.54	57,476.3	58,002.2	0.91%	29,349	29,245.1	99.65%

**Table 10:** Impact of using different levels of probability for Scenario 2.

Scenario 2	*O*	$O_{c}$	*T*	$T_{v}$	*S*	$S_{c}$	*R*	end	avg	*C*	$\bar{C}$	*G*(%)	*E*	*V*	*V*(%)
[0%,33%]	14,846	1730	1502	221	13,336	1501	8	7199.98	7187.96	57,503.7	59,230.9	3.00%	30,599	27,098.7	88.56%
[33%,66%]	8425	157	2204	97	6213	54	8	7199.66	7172.36	57,378.8	58,146.6	1.34%	30,599	30,267	98.91%
[66%,100%]	5486	60	2457	53	3021	0	8	7199.88	7188.25	57,506	58,016	0.89%	30,599	30,481.2	99.62%

**Table 11:** Impact of using different levels of probability for Scenario 3.

Scenario 3	*O*	$O_{c}$	*T*	$T_{v}$	*S*	$S_{c}$	*R*	end	avg	*C*	$\bar{C}$	*G*(%)	*E*	*V*	*V*(%)
[0%,33%]	15,046	1836	1446	230	13,592	1599	8	7199.89	7170.32	57,362.5	59,323.2	3.42%	31,711	28,032	88.40%
[33%,66%]	8638	153	2204	103	6426	43	8	7199.75	7174.39	57,395.1	58,243.2	1.48%	31,711	31,339.3	98.83%
[66%,100%]	5545	80	2438	69	3099	1	8	7199.6	7180.72	57,445.8	58,156.1	1.24%	31,711	31,597.5	99.64%

**Table 12:** Results varying the number of expected victims for Scenario 1.

Scenario 1	*O*	$O_{c}$	*T*	$T_{v}$	*S*	$S_{c}$	*R*	end	avg	*C*	$\bar{C}$	*G*(%)	*E*	*V*	*V*(%)
**<50–75%**	6422	318	2311	87	4103	224	8	7199.81	7178.59	57,428.7	58,234.6	1.40%	11,036	10,977	99.47%
**<25–50%**	6757	186	2359	78	4390	101	8	7199.41	7183.31	57,466.5	58,187.1	1.25%	18,400	18,300.7	99.46%
**<0–25%**	6387	155	2344	118	4035	30	8	7199.51	7166.16	57,329.3	58,504.6	2.05%	25,763	25,540.7	99.14%
**Initial Scenario**	6592	300	2317	74	4267	221	8	7199.71	7194.58	57,556.608	58,383.042	1.44%	29,349	29,200	99.49%
**>0–25%**	6474	174	2371	61	4095	107	8	7199.8	7172.94	57,383.6	57,969.9	1.02%	33,018	32,826.2	99.42%
**>25–50%**	6677	158	2310	113	4359	38	8	7187.24	7174.7	57,397.6	58,623.2	2.14%	40,385	40,116.8	99.34%
**>50–75%**	6638	232	2352	76	4278	150	8	7199.98	7190.77	57,526.2	58,247	1.25%	47,749	47,535.1	99.55%

**Table 13:** Results varying the number of expected victims for Scenario 2.

Scenario 2	*O*	$O_{c}$	*T*	$T_{v}$	*S*	$S_{c}$	*R*	end	avg	*C*	$\bar{C}$	*G*(%)	*E*	*V*	*V*(%)
**<50–75%**	6070	130	2372	97	3690	26	8	7199.24	7157.72	57,261.8	58,109.9	1.48%	11390	11,328.2	99.46%
**<25–50%**	6121	71	2408	52	3705	13	8	7199.85	7186.76	57,494.1	58,046.4	0.96%	19,080	18,967.5	99.41%
**<0–25%**	6147	95	2370	77	3769	11	8	7199.41	7177.1	57,416.8	58,166.4	1.31%	26,746	26,605.7	99.48%
**Initial Scenario**	6167	127	2380	59	3779	61	8	7199.74	7184.47	57,475.8	58,147.7	1.17%	30,599	30,439.6	99.48%
**>0–25%**	6075	125	2376	81	3691	37	8	7199.67	7177.93	57,423.5	58,215.3	1.38%	34,309	34,105	99.41%
**>25–50%**	6127	73	2409	59	3710	8	8	7199.97	7189.97	57,519.8	58,106.4	1.02%	41,989	41,749.5	99.43%
**>50–75%**	6216	82	2431	64	3777	12	8	7199.87	7192.26	57,538	58,123.3	1.02%	49,679	49,394.8	99.43%

**Table 14:** Results varying the number of expected victims for Scenario 3.

Scenario 2	*O*	$O_{c}$	*T*	$T_{v}$	*S*	$S_{c}$	*R*	end	avg	*C*	$\bar{C}$	*G*(%)	*E*	*V*	*V*(%)
**<50–75%**	6325	288	2356	71	3961	211	8	7199.99	7176.97	57,415.7	58,155.7	1.29%	11,837	11,775	99.48%
**<25–50%**	6434	336	2342	79	4084	250	8	7199.77	7182.4	57,459.2	58,187	1.27%	19,795	19,686.4	99.45%
**<0–25%**	6443	129	2375	75	4060	48	8	7199.81	7186.02	57,488.1	58,100.4	1.07%	27,755	27,569	99.33%
**Initial Scenario**	6634	115	2378	87	4248	20	8	7190.95	7187.29	57,498.4	58,299.2	1.39%	31,711	31,544.3	99.47%
**>0–25%**	6742	107	2344	70	4390	32	8	7199.99	7172.69	57,381.5	57,998.6	1.08%	35,604	35,382.9	99.38%
**>25–50%**	6717	72	2379	51	4330	14	8	7199.83	7190.5	57,524	57,999.2	0.83%	43,548	43,303.1	99.44%
**>50–75%**	6862	98	2322	62	4532	28	8	7199.16	7177.8	57,422.4	58,010.3	1.02%	51,506	51,234.4	99.47%

**Table 15:** Results for the underestimate and overestimate cases using Scenario 1.

Scenario 1	*O*	$O_{c}$	*T*	$T_{v}$	*S*	$S_{c}$	*R*	end	avg	*C*	$\bar{C}$	*G*(%)	*E*	*V*	*V*(%)
**<15–20%**	6512	170	2377	113	4127	113	8	7199.69	7189.60	57,516.80	58,105.50	1.02%	29,349	28,565.40	97.33%
**<10–15%**	6522	198	2350	66	4164	126	8	7199.73	7183.69	57,469.5	58,153.7	1.19%	29,349	28,824.5	98.21%
**<5–10%**	6528	221	2310	78	4210	137	8	7199.83	7170.27	57,362.10	58,134.5	1.35%	29,349	28,980.90	98.75%
**<0–5%**	6518	243	2341	92	4169	144	8	7199.50	7180.63	57,445	58,329.30	1.54%	29,349	29,099	99.15%
**>0–5%**	6657	108	2343	74	4306	28	8	7199.50	7181.69	57,453.50	58,240.20	1.37%	29,349	29,221.50	99.57%
**>5–10%**	5761	415	2352	117	3401	291	8	7199.71	7187.25	57,498	58,618.4	1.95%	29,349	29,239.5	99.63%
**>10–15%**	5709	205	2410	67	3291	131	8	7199.58	7183.81	57,470.5	58,165.1	1.21%	29,349	29,260.1	99.70%
**>15–20%**	5441	165	2445	53	2988	107	8	7199.94	7187.14	57,497.1	58,098.2	1.05%	29,349	29,277.2	99.76%

**Table 16:** Results for the underestimate and overestimate cases using Scenario 2.

Scenario 2	*O*	$O_{c}$	*T*	$T_{v}$	*S*	$S_{c}$	*R*	end	avg	*C*	$\bar{C}$	*G*(%)	*E*	*V*	*V*(%)
**<15%–20%**	6144	152	2384	71	3752	73	8	7199.73	7195.32	57,562.6	58,282.6	1.25%	30,599	29,829.8	97.49%
**<10–15%**	6241	140	2368	81	3865	52	8	7199.21	7174.03	57,392.3	58,203.5	1.41%	30,599	30,091.4	98.34%
**<5–10%**	6363	109	2397	43	3958	59	8	7199.84	7189.18	57,513.5	57,996	0.84%	30,599	30,283.5	98.97%
**>0–5%**	6157	87	2400	57	3749	23	8	7199.85	7172.55	57,380.4	57,968.8	1.03%	30,599	30,377.9	99.28%
**>0–5%**	5994	111	2400	54	3586	51	8	7199.86	7186.83	57,494.6	58,040.5	0.95%	30,599	30,458.3	99.54%
**>5–10%**	5690	67	2441	53	3241	6	8	7199.32	7185.76	57,486.1	58,042.5	0.97%	30,599	30,476.8	99.60%
**>10–15%**	5381	198	2474	69	2899	122	8	7199.62	7194.15	57,553.2	58,284.6	1.27%	30,599	30,496.8	99.67%
**>15–20%**	5235	99	2452	49	2775	43	8	7199.83	7192.64	57,541.1	58,042	0.87%	30,599	30,539	99.80%

**Table 17:** Results for the underestimate and overestimate cases using Scenario 3.

Scenario 3	*O*	$O_{c}$	*T*	$T_{v}$	*S*	$S_{c}$	*R*	end	avg	*C*	$\bar{C}$	*G*(%)	*E*	*V*	*V*(%)
**<15–20%**	6630	83	2377	67	4245	11	8	7199.93	7184.34	57,474.7	58,055.8	1.01%	31,711	30,797.8	97.12%
**<10–15%**	6370	378	2359	70	4003	303	8	7199.9	7176.78	57,414.2	58,095.8	1.19%	31,711	31,076.2	98.00%
**<5–10%**	6666	80	2395	60	4263	14	8	7199.69	7195.88	57,567	58,074.1	0.88%	31,711	31,315.3	98.75%
**<0–5%**	6660	115	2366	80	4286	28	8	7199.91	7188.45	57,507.6	58,214.8	1.23%	31,711	31,484.2	99.28%
**<0–5%**	6571	92	2391	62	4172	24	8	7199.87	7192	57,536	58,130	1.03%	31,711	31,536.8	99.45%
**>5–10%**	5961	103	2444	75	3509	20	8	7199.91	7189.32	57,514.5	58,232.7	1.25%	31,711	31,564.2	99.54%
**>10–15%**	5740	299	2426	60	3306	232	8	7199.56	7191.34	57,530.7	58,139.4	1.06%	31,711	31,604.4	99.66%
**>15–20%**	5511	79	2482	58	3021	13	8	7199.77	7185.79	57,486.3	57,981.4	0.86%	31,711	31,631.2	99.75%

## Data Availability

Data are contained within the article.

## Abbreviations

The following abbreviations are used in this manuscript:

ALNS	Adaptive Large Neighborhood Search
CL	Candidate List
CBBA	Consensus-Based Bundle Algorithm
DA	Drone Agent
DRP	Drone Routing Problems
DSH	Drone Swarm Heuristic
DSRP	Drone Swarms Routing Problem
ILP	Integer Linear Programming
MC	Main Control
MILP	Mixed-Integer Linear Programming
MINLP	Mixed-Integer Nonlinear Programming
MBATA	Modified Backtracking Adaptive Threshold Accepting
MAS	Multi-Agents Systems
MRTA	Multi-Robot Task Allocation
OSM	OpenStreetMap
VRP	Vehicle Routing Problem
